# The endoplasmic reticulum in plant immunity and cell death

**DOI:** 10.3389/fpls.2012.00200

**Published:** 2012-08-22

**Authors:** Ruth Eichmann, Patrick Schäfer

**Affiliations:** The School of Life Sciences, University of WarwickCoventry, UK

**Keywords:** programed cell death, plant immunity, unfolded protein response, stress, endoplasmic reticulum quality control

## Abstract

The endoplasmic reticulum (ER) is a highly dynamic organelle in eukaryotic cells and a major production site of proteins destined for vacuoles, the plasma membrane, or apoplast in plants. At the ER, these secreted proteins undergo multiple processing steps, which are supervised and conducted by the ER quality control system. Notably, processing of secreted proteins can considerably elevate under stress conditions and exceed ER folding capacities. The resulting accumulation of unfolded proteins is defined as ER stress. The efficiency of cells to re-establish proper ER function is crucial for stress adaptation. Besides delivering proteins directly antagonizing and resolving stress conditions, the ER monitors synthesis of immune receptors. This indicates the significance of the ER for the establishment and function of the plant immune system. Recent studies point out the fragility of the entire system and highlight the ER as initiator of programed cell death (PCD) in plants as was reported for vertebrates. This review summarizes current knowledge on the impact of the ER on immune and PCD signaling. Understanding the integration of stress signals by the ER bears a considerable potential to optimize development and to enhance stress resistance of plants.

## INTRODUCTION

The endoplasmic reticulum (ER) is an organelle with important functions in eukaryotic cells. It connects to other cellular compartments [e.g., nucleus, Golgi apparatus, mitochondria, peroxisomes, plasma membrane (PM)] and, as one of the largest Ca^2+^ stores, participates in intracellular Ca^2+^ signaling. It is further involved in lipid and hormone biosynthesis (Staehelin, 1997; Sparkes et al., 2009; Lynes and Simmen, 2011). Importantly, the ER quality control (ER-QC) system mediates and monitors the processing and folding of secretory proteins destined for transport to the PM, vacuole, or apoplast, identifies misfolded proteins and transfers them to the ER-associated degradation (ERAD) machinery (Vitale and Boston, 2008; Liu and Howell, 2010; Hüttner and Strasser, 2012). Among the proteins processed by the plant’s ER-QC are important PM-resident proteins involved in adaptation to environmental stress, e.g., hormone or immune receptors (Saijo, 2010). ER integrity is central to proper function of cells and whole organisms. Especially under stress conditions, any impairment of ER function can result in disturbed plant development and plant immunity (Wang et al., 2005; Vitale and Boston, 2008; Saijo, 2010).

## REGULATION OF ER INTEGRITY AND ER STRESS SIGNALING IN EUKARYOTES

Protein folding demand and capacities in the ER are usually in equilibrium. However, responses to environmental stresses create an increased requirement for secreted proteins. If this demand exceeds the ER-QC working capacity, unfolded proteins accumulate in the ER, which the cell senses as ER stress. Prolonged ER stress impairs ER function and thus threatens cellular integrity. Chemicals, such as the *N*-glycosylation inhibitor tunicamycin (TM) or the reducing agent dithiothreitol (DTT), which inhibits the formation of disulfide bonds, are widely used to induce and examine ER stress (Martínez and Chrispeels, 2003; Kamauchi et al., 2005; Vitale and Boston, 2008; Liu and Howell, 2010).

**FIGURE 1:**
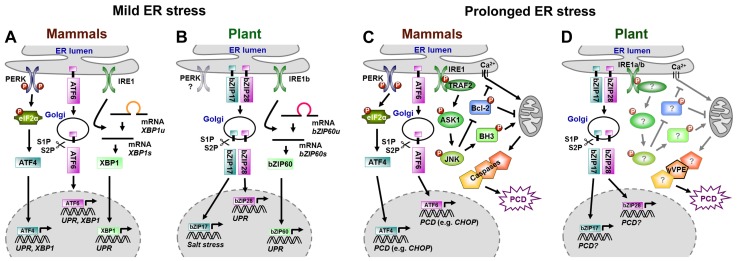
**Signaling in mammals and plants under mild (A,B) and prolonged ER stress (C,D)**. Models indicate overlaps and differences in ER stress signaling. Conservation in mammalian **(A)** and plant **(B)** UPR signaling in response to mild ER stress. Various components involved in mammalian ER-PCD signaling under prolonged ER stress have been identified **(C)**, whereas plant ER-PCD signaling is almost unknown **(D)**. Question marks (in **D**) indicate postulated orthologs or structural homologs of plant ER-PCD signaling. XBP1u/bZIP60u, unspliced mRNA; XBP1s/bZIP60s, spliced mRNA.

In animals, mainly three ER membrane proteins constitute the cell’s ER stress surveillance system: the type I transmembrane protein kinase/endoribonuclease inositol-requiring enzyme 1 (IRE1 α and β), the type I transmembrane protein kinase RNA-like ER kinase (PERK), and the type II transmembrane basic leucine-zipper (bZIP) domain-containing activating transcription factor 6 (ATF6). In yeast cells, IRE1 is the only ER stress sensor (Mori, 2009). Under non-stressed conditions, luminal parts of these ER stress sensors bind to luminal binding proteins (BiPs), which keeps the sensors in an inactive state. If unfolded proteins accumulate, BiPs disconnect from ER stress sensors to mediate processing of unfolded proteins. Once liberated, ER stress sensors initiate different adaptive signaling cascades defined as unfolded protein response (UPR) to re-establish proper ER function. The UPR enhances the synthesis of antioxidants and ER-QC members, attenuates translation, suppresses expression of secretory genes, and elevates ERAD of unfolded proteins (Schröder, 2006, 2008; Liu and Howell, 2010; Hetz, 2012;Higa and Chevet, 2012; Jäger et al., 2012). Figure 1A summarizes processes involved in UPR activation by the three ER stress sensors in animals. BiP release allows ATF6 translocation to the Golgi apparatus, where its cytosolic part (cATF6), a functional bZIP transcription factor, is cleaved off by serine proteases S1P and S2P, a process called regulated intramembrane proteolysis (RIP). cATF6 then enters the nucleus and promotes transcription of *UPR* genes and the bZIP transcription factor *XBP1* (Yoshida et al., 2001). Upon BiP release, IRE1 oligomerizes and activates its endoribonuclease domain, leading to the unconventional splicing of a 26 nucleotide intron out of *XBP1* or its yeast counterpart *HAC1*, which allows the resulting proteins to enter the nucleus (Mori, 2009; Walter and Ron, 2011; Hetz, 2012). Phosphorylation by the PERK kinase activates the eukaryotic translation initiation factor eIF2α, which attenuates translation but selectively promotes the translation of the transcription factor ATF4 (Harding et al., 2000). Eventually, ATF4, ATF6, and XBP1 (HAC1) elevate transcription of *UPR* genes (Mori, 2009; Walter and Ron, 2011; Hetz, 2012).

In plants, the ER-QC and ER stress responses are apparently conserved as suggested by sequence homologies found in *Arabidopsis* for members of the ER translocon and oligosaccharyltransferase complexes as well as for UPR and ERAD components (Liu and Howell, 2010). Further, transcripts of genes encoding proteins of the ER-QC machinery [e.g., chaperones BiPs, CALRETICULINs (CRTs), CALNEXINs (CNXs) or PROTEIN DISULFIDE ISOMERASEs (PDIs)], or the ERAD pathway are induced by ER stress (Jelitto-Van Dooren et al., 1999; Leborgne-Castel et al., 1999; Koizumi et al., 2001; Martínez and Chrispeels, 2003; Kamauchi et al., 2005; Lu and Christopher, 2008; Su et al., 2011; Hüttner and Strasser, 2012). Putative plant ER stress sensors and signaling components have been identified (Figure 1B), however, except for IRE, respective plant proteins do not show sequence but structural or functional homology (Koizumi et al., 2001; Liu and Howell, 2010). *Arabidopsis* possesses at least two IRE1-like proteins, while only one homolog is present in rice (*Oryza sativa*). AtIRE1a, AtIRE1b, and OsIRE1 harbor all structural features of yeast and mammalian IRE1. AtIRE1a and OsIRE1 are capable of autotransphosphorylation, and the putative ER stress sensor domain of AtIRE1a, AtIRE1b, and OsIRE1 can functionally replace that of yeast IRE1 (Koizumi et al., 2001; Noh et al., 2002; Okushima et al., 2002). There are at least three ER-resident transmembrane bZIP transcription factors in *Arabidopsis*, which are involved in ER stress responses, AtbZIP17, AtbZIP28, and AtbZIP60 (Urade, 2009; Liu and Howell, 2010). *Atbzip* mutants do not display morphological or developmental differences under non-stress conditions, but are more sensitive to salt stress (*Atbzip17*, Liu et al., 2007b), heat (*Atbzip28*, Gao et al., 2008), or DTT treatment (*Atbzip60*, Humbert et al., 2012). The expression of salt stress responsive genes is impaired in *Atbzip17* mutants (Liu et al., 2007b) as is the induction of canonical *UPR* genes in *Atbzip28* and *Atbzip60* mutants after TM treatment (Iwata and Koizumi, 2005a; Liu et al., 2007a; Iwata et al., 2008; Lu and Christopher, 2008; Tajima et al., 2008). Similar to ATF6 in mammals, AtbZIP17 and AtbZIP28 possess canonical S1P cleavage sites and are activated by a RIP-like process upon ER stress (Liu et al., 2007a,b, 2008a; Gao et al., 2008; Tajima et al., 2008; Che et al., 2010). RIP of AtbZIP17 and AtbZIP28 requires passage through the Golgi apparatus, where cleavage by the subtilisin-like serine protease AtS1P and subsequent processing by the metalloprotease AtS2P take place (Liu et al., 2007a,b; Che et al., 2010; Srivastava et al., 2012). How these bZIPs sense ER stress and how Golgi transition is mediated, is not clear. However, TM treatment apparently promotes the interaction of AtbZIP28 with the small GTPase SAR1b and the guanidine exchange factor SEC12, which are putatively involved in coat protein complex II (COPII) vesicle formation during ER-to-Golgi transport (Srivastava et al., 2012). AtbZIP60 lacks a canonical S1P cleavage site and its activation is independent of S1P and S2P (Iwata et al., 2008). Similar to mammalian XBP1 and yeast HAC1, recent studies in *Arabidopsis* and rice revealed unconventional splicing of a 23 nucleotide intron from the *AtbZIP60* mRNA by AtIRE1b or AtIRE1a, and a 20 nucleotide intron from its rice ortholog*OsbZIP50/OsbZIP74* mRNA by OsIRE1, e.g., after TM or salicylic acid (SA) treatment. This leads to a frame shift that removes the transmembrane domain of the new proteins and allows nuclear entrance (Deng et al., 2011; Nagashima et al., 2011; Hayashi et al., 2012; Humbert et al., 2012; Lu et al., 2012; Moreno et al., 2012). There are no obvious PERK homologs in *Arabidopsis* (Koizumi et al., 2001; Urade, 2009).

## ER STRESS AS INITIATOR OF PROGRAMED CELL DEATH

The UPR is supposed to ensure cell survival. However, under prolonged or severe ER stress, mammalian cells activate an apoptosis-like programed cell death (ER-PCD) to eliminate damaged cells from stressed organisms (Schröder, 2006; Hetz, 2012; Jäger et al., 2012). The ER stress sensors ATF6, PERK, and IRE1 are central regulators of this process as well (Figure 1C), although it is unclear how they perceive and differentiate signals to switch from UPR to apoptosis. ER-PCD obviously merges with other apoptosis pathways, involving enhanced generation of reactive oxygen species (ROS), and apoptosis-promoting Ca^2+^ signaling at ER and mitochondria (Chakrabarti et al., 2011; Gorman et al., 2012; Hetz, 2012; Jäger et al., 2012). The induction of the pro-apoptotic bZIP transcription factor CHOP (C/EBP-homologs protein) by ATF6 and PERK/ATF4 during ER-PCD apparently is most relevant. CHOP down-regulates anti-apoptotic proteins (e.g., BCL-2), but induces members of the pro-apoptotic (BH3)-only protein family, e.g., BIM (BCL-2-INTERACTING MEDIATOR OF CELL DEATH) or GADD34 (GROWTH ARREST AND DNA DAMAGE-INDUCIBLE 34; Gorman et al., 2012; Hetz, 2012; Jäger et al., 2012). In addition, IRE1 activates ER-PCD by interacting with TRAF2 (TUMOR NECROSIS FACTOR RECEPTOR-ASSOCIATED FACTOR 2; Gorman et al., 2012; Jäger et al., 2012). This initiates consecutive phosphorylation of ASK1 (APOPTOSIS SIGNAL-REGULATING KINASE 1) and JNK (JUN N-TERMINAL KINASE). Phosphorylation by JNK inactivates anti-apoptotic regulators such as BCL-2, but activates pro-apoptotic BH3-only proteins such as BIM or BID (BH3-interacting domain death agonist). BH3-only proteins promote the cell death activation-related oligomerization and translocation of BAX and BAK to the mitochondrial membrane, followed by cytochrome *c* release and caspase activation for execution of apoptosis. BCL-2-dependent regulation of Ca^2+^ homeostasis of the ER also affects permeability transition and apoptosis signaling at mitochondria (Chakrabarti et al., 2011; Gorman et al., 2012; Hetz, 2012). BAX and BAK themselves can interact with IRE1 and promote its ability to activate ASK1 and JNK, processes that are apparently blocked by the cell survival protein BI-1 (BAX INHIBITOR-1; Bailly-Maitre et al., 2009; Lisbona et al., 2009). Dynamic differential interactions with pro- and anti-apoptotic proteins modulated by the intensity and duration of ER stress signals might regulate separate functions of IRE1, and timely coordinated on- and offset of ATF6, PERK, and IRE1 signaling may play a decisive role in determining cell fate. In such a scenario, ER stress would initially activate the adaptive UPR via IRE1-mediated splicing of XBP1. However, down-regulation of the IRE1/XBP1 branch upon prolonged ER stress may give rise to pro-apoptotic IRE1/TREF2/ASK1/JNK, RIDD, and/or PERK signaling (Gorman et al., 2012; Hetz, 2012). Autophagy is further suggested to abolish ER stress in yeast and mammals as it might support the removal of unfolded proteins (Bernales et al., 2006). Here, the PERK-elF2α-ATF4 and IRE/TRAF2/JNK pathways might connect autophagy to ER stress via the BECLIN1-BCL2 interaction and the induction of autophagy genes, respectively. Although ER stress-associated autophagy is thought to have a cytoprotective function, other studies suggest a role in ER-PCD. However, regulators of this cell death pathway and its link to ER stress are currently unknown (Verfaillie et al., 2010; Aronson and Davies, 2012).

As in animal cells, cell death follows induction of UPR in TM-treated plants (Zuppini et al., 2004; Iwata and Koizumi, 2005b; Watanabe and Lam, 2008; Ishikawa et al., 2011). The molecular basis of plant ER-PCD and the role of plant bZIPs therein are largely unknown (Figure 1D). However, regulation of ER-PCD seems to be partially conserved across kingdoms, as *Arabidopsis* BI-1 (AtBI-1) is involved in restriction of ER-PCD in *Arabidopsis* as well (Watanabe and Lam, 2008; Ishikawa et al., 2011). *AtBI-1* is AtbZIP60-dependently up-regulated in response to TM (Kamauchi et al., 2005; Iwata et al., 2008; Watanabe and Lam, 2008). AtBI-1-mediated inhibition of ER-PCD in *Arabidopsis* is likely un-related to UPR modification, but rather to the suppression of ER-dependent ROS production or regulation of cell death associated ER Ca^2+^ homeostasis (Watanabe and Lam, 2008, 2009). In *Arabidopsis*, a Gβ subunit of an ER-resident heterotrimeric GTP-binding protein, AGB1, might be involved in the promotion of ER-PCD (Wang et al., 2007; Chen and Brandizzi, 2012). Disturbed ER protein retention after silencing of *NbERD2a*/*NbERD2b* interferes with ER-QC and reduces ER stress alleviation, resulting in enhanced PCD in response to bacterial pathogens (Xu et al., 2012). New insights into the role of vacuolar processing enzymes with caspase1-like activities in the execution of ER-PCD come from Qiang et al. (2012). These studies demonstrate the dependence of the mutualistic fungus *Piriformospora indica* on ER-PCD for successful *Arabidopsis* root colonization. *P. indica* induces ER stress but suppresses the adaptive UPR pathway. Consequently, the *P. indica*-induced ER stress triggers a vacuolar cell death pathway whose execution depends on γ VACUOLAR PROCESSING ENZYME (γVPE). This ER-PCD can be phenocopied by the application of TM to *Arabidopsis* roots. The analyses further show that γVPE is responsible for enhanced VPE and caspase 1-like activities during TM- and *P. indica*-induced ER-PCD (Qiang et al., 2012).

## ER – EXECUTOR OF PLANT IMMUNITY AND PUTATIVE TARGET OF PATHOGEN EFFECTORS

Plants ward off pathogens by a multi-layered immune system. PM localized pattern recognition receptors (PRRs) detect conserved molecules, so-called microbe-associated molecular patterns (MAMPs), of invading microbes. Well-characterized PRRs are FLAGELLIN-SENSING 2 (FLS2), which recognizes bacterial flagellin, the ELONGATION-FACTOR TU (EF-Tu) RECEPTOR (EFR), which detects bacterial EF-Tu, and the chitin receptors CHITIN ELICITOR BINDING PROTEIN (CEBiP) and CHITIN ELICITOR RECEPTOR KINASE (CERK; Monaghan and Zipfel, 2012). MAMP perception by these PRRs initiates immune signaling pathways, defined as MAMP-triggered immunity (MTI), which involve Ca^2+^ fluxes across the PM, a rapid production of ROS, the activation of mitogen-activated protein kinase cascades and WRKY transcription factors, eventually resulting in the induction of defense mechanisms including callose depositions and the synthesis of antimicrobial pathogenesis-related (PR) proteins (Jones and Dangl, 2006; Boller and Felix, 2009). Successful pathogens have evolved effector molecules to suppress MTI. Plant RESISTANCE (R) proteins specifically recognize pathogen effectors or their activities and initiate effector-triggered immunity (ETI), typically involving hypersensitive response (HR)-related PCD (Chisholm et al., 2006; Jones and Dangl, 2006). The ER participates in plant innate immunity in several ways. Firstly, immunity depends on the secretory apparatus for the production of immune proteins (Wang et al., 2005; Nekrasov et al., 2009; Saijo et al., 2009). NONEXPRESSOR OF PR GENES 1 (NPR1), the master regulator of SA-dependent systemic acquired resistance (SAR), coordinately controls the up-regulation of *PR* genes and genes encoding proteins of the secretory pathway during SAR (Wang et al., 2005). Secondly, synthesis and proper function of PRRs (e.g., EFR) rely on *N*-glycosylation and the ER-QC system, which involves staurosporine and temperature sensitive-3a (STT3A), glucosidase II, the H/KDEL receptor ERD2b, the UDP-glucose:glycoprotein glucosyltransferase (UGGT)/CRT3 cycle and the stromal cell-derived factor-2 (SDF2)/ERdj3B/BiP complex (Li et al., 2009; Lu et al., 2009; Nekrasov et al., 2009; Saijo et al., 2009; Saijo, 2010). Susceptibility of ER-QC mutants to pathogens differs qualitatively and quantitatively from that of *efr* mutants, suggesting the existence of EFR-independent but ER-QC-dependent immune response (Li et al., 2009; Nekrasov et al., 2009; Saijo et al., 2009). Meanwhile, a number of membrane-localized immune receptors have been identified, whose functions depend on ER-QC, among them the rice PRR XA21 involved in resistance to *Xanthomonas oryzae* pv. *oryzae* (Park et al., 2010a,b), an induced receptor kinase (IRK), which is involved in *N*-mediated resistance of tobacco to tobacco mosaic virus (Caplan et al., 2009), and glycosylated Cf proteins, which confer race-specific resistance to the fungal pathogen *Cladosporium fulvum* (Liebrand et al., 2012). Similar to FLS2, the ER-QC disturbance does not affect CERK1 function in *Arabidopsis* (Li et al., 2009; Nekrasov et al., 2009). However, the rice homolog OsCERK1 seems to interact with a Hop/Sti1-Hsp90 chaperone complex for maturation in the ER prior to transport to the PM (Chen et al., 2010). ER-QC also monitors glycosylation and proper folding of some immunity-related Toll-like receptors (TLRs) that recognize MAMPs in animals (Yang et al., 2007). Interestingly, PRRs TLR4 and TLR2 activate the IRE1α-XBP1 pathway to enhance secretion of certain proinflammatory cytokines in macrophages, and loss of XBP1 function impairs immunity against the bacterial pathogen *Francisella tularensis* (Martinon et al., 2010).

Induction of the ER-QC machinery accompanies synthesis of immunity-associated proteins in plants (Jelitto-Van Dooren et al., 1999; Wang et al., 2005). Consequently, ER-QC mutants are more susceptible to ER stress inducers and pathogens (Wang et al., 2005; Li et al., 2009; Lu et al., 2009; Nekrasov et al., 2009; Saijo et al., 2009). Similarly, proper execution of defense responses may rely on the induction of *UPR* genes. Recently, the heat-shock factor-like transcription factor TBF1 has been identified as important transcriptional regulator of *UPR* genes, and *Arabidopsis tbf1* mutants are impaired in the execution of SAR and EFR-mediated MTI (Pajerowska-Mukhtar et al., 2012). The *Nicotiana benthamiana* homolog of *AtbZIP60*, *NbbZIP60*, is induced in response to inoculation with avirulent *Pseudomonas cichorii* and required to arrest its growth (Tateda et al., 2008). Furthermore, *AtIRE1a* and *AtIRE1b* expression is pathogen-responsive, and both proteins are required for SA or pathogen-dependent splicing of *AtbZIP60*, expression of ER-QC genes, secretion of defense proteins and thus execution of SAR (Moreno et al., 2012).

Together, this underlines the functional importance of the ER in both MTI and ETI, and designates it as a potential effector target. Consistent with this, many viruses employ host UPR by targeting ER stress sensors to enhance folding of viral proteins or to modulate immune responses in mammals (Ke and Chen, 2011; Qian et al., 2012). In tobacco, infection with *Potato virus X* or overexpression of a viral movement protein induces *bZIP60* and *UPR* genes possibly to suppress host cell death responses (Ye et al., 2011). In addition, Yamamoto et al. (2011) showed that ATF6β is part of mice immunity against the protozoan parasite *Toxoplasma gondii*. ROP18, a serine/threonine kinase, which is secreted into the host cell during infection, interacts with ATF6β and mediates its proteasome-dependent degradation. Thus, ATF6β constitutes a target for the *T. gondii* ROP18 virulence factor possibly to suppress UPR-mediated host defense. Likewise, the *Salmonella enterica* leucine-rich repeat (LRR) effector protein SlrP targets the host ER-QC member ERdj3. This supports infection as it leads to the accumulation of unfolded proteins eventually promoting host cell death (Bernal-Bayard et al., 2010). In *Caenorhabditis elegans*, the increased requirement of secreted proteins during the activation of immune responses imposes ER stress to the organism itself, which requires XBP1-mediated UPR to avoid onset of ER-PCD (Richardson et al., 2010). Several bacterial toxins, e.g., Shiga toxin produced by enterohemorrhagic bacteria, can enter the ER and seem to initiate cell death through prolonged UPR signaling by activating ER stress sensors (Tesh, 2012).

## CONCLUSIONS AND PERSPECTIVE

As production site of antimicrobial proteins and of immune signaling components, the ER functions as central regulator in the execution of immune responses in plants and animals. Therefore, the disturbance of ER integrity is certainly of primary relevance for pathogens to achieve host cell infection. Plants further rely on proper ER function and likely ER membrane localized stress sensors for adaptation to abiotic stress such as salt or heat stress (Liu et al., 2008a,b, 2011; Che et al., 2010; Cui et al., 2012). Taken together, the improvement of plant UPR in order to maintain ER homeostasis under unfavorable conditions may increase plant adaptability to biotic and abiotic stress, which bears a potential to enhance crop yield and yield stability.

## Conflict of Interest Statement

The authors declare that the research was conducted in the absence of any commercial or financial relationships that could be construed as a potential conflict of interest.
